# Supplementary material for: Quantifying tourism booms and the increasing footprint in the Arctic with social media data
Source: PLoS One. 2020 Jan 16;15(1):e0227189. doi: 10.1371/journal.pone.0227189 (PMC6964912; doi:10.1371/journal.pone.0227189)
Supplement: S1 Appendix — (PDF) [file pone.0227189.s001.pdf]

Appendix S1. Sensitivity analysis of photo exclusion threshold

The median number of photos submitted to Flickr by each user ranged across countries from 25-33 photos. At the front end of this distribution are a set of users who submitted only 1 or 2 photos to the platform. These ‘test users’ account for approximately a third of users but only a tiny fraction of photos. When we examined the photographs of a sample of these test users they appeared to be ‘random snaps’ i.e. out of focus or with no discernible subject. For this reason we excluded the photographs of these users from our dataset. We chose a threshold of 2 photos to balance retaining quality photos without excluding too many users. People contributing 10 or fewer photos account for less than 1% of the number of photographs, but over two thirds of the users. This dropped to one third of users with 2 or fewer photos.

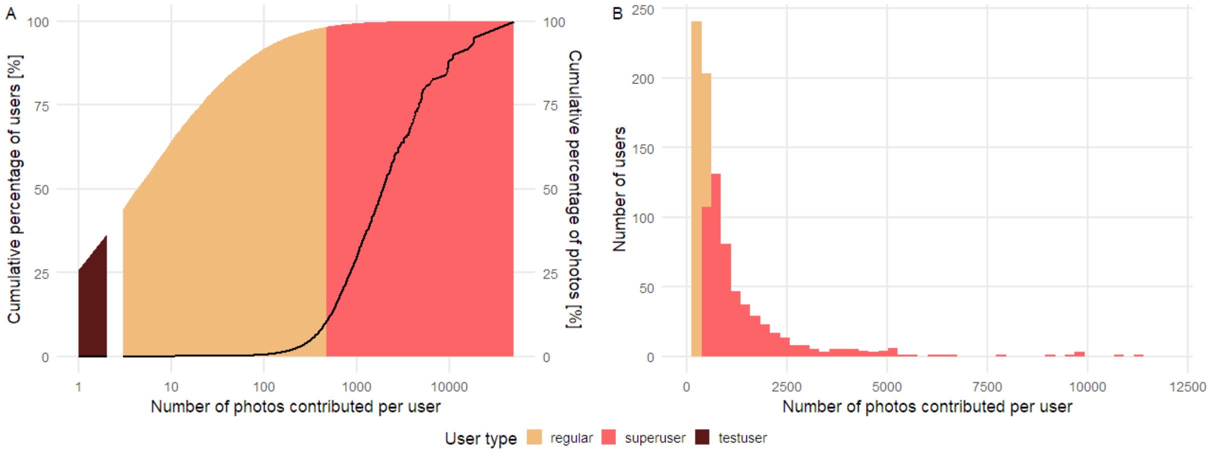

Figure S1. A. Cumulative plots, by number of photographs contributed per user, of the percentage of users (coloured fill) and percentage of the total number of photos (black line) submitted to Flickr in the Arctic. The x axis is on log scale. B. shows a histogram of the number photos contributed per user.
