# Supplementary material for: Quantifying tourism booms and the increasing footprint in the Arctic with social media data
Source: PLoS One. 2020 Jan 16;15(1):e0227189. doi: 10.1371/journal.pone.0227189 (PMC6964912; doi:10.1371/journal.pone.0227189)
Supplement: S2 Appendix — (PDF) [file pone.0227189.s002.pdf]

S2 Appendix: Sensitivity analysis of resolution

Sensitivity analysis using square raster cells indicates that for the footprint analysis, 5 km diameter hexagons (21.6 km^2 cell area) is an appropriate compromise between commission errors (overestimating footprint) and computing efficiency. For temporal trend estimates, 10 km diameter hexagonal cells (86.6 km^2 cell area) seems a good compromise between providing spatial resolution at a scale relevant for management and having sufficient data in each cell to analyse trends.

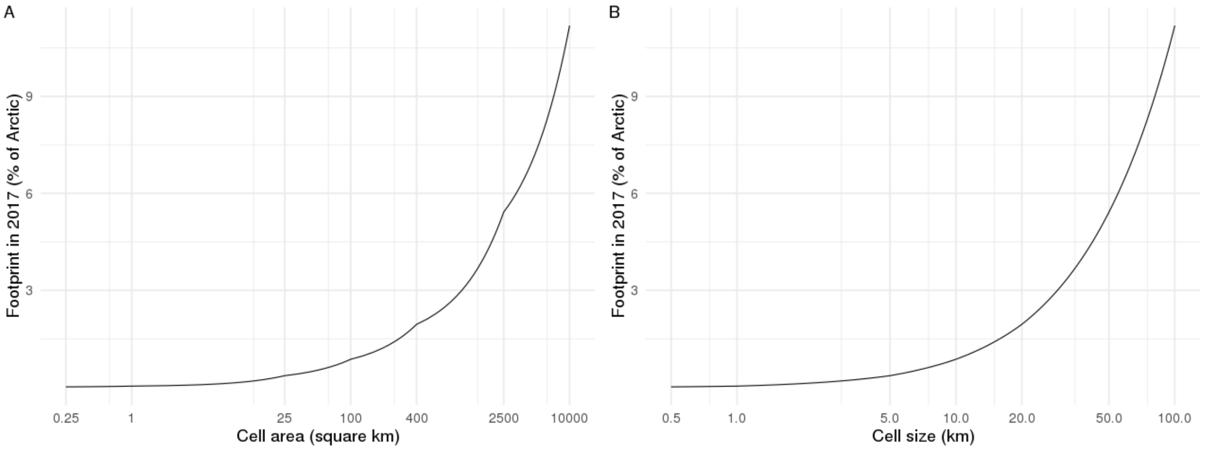

Figure S2.1 Tourism footprint estimated from Flickr data relative to the resolution of the raster at which point data was aggregated A. Cell area (km) B. Cell diameter (km).

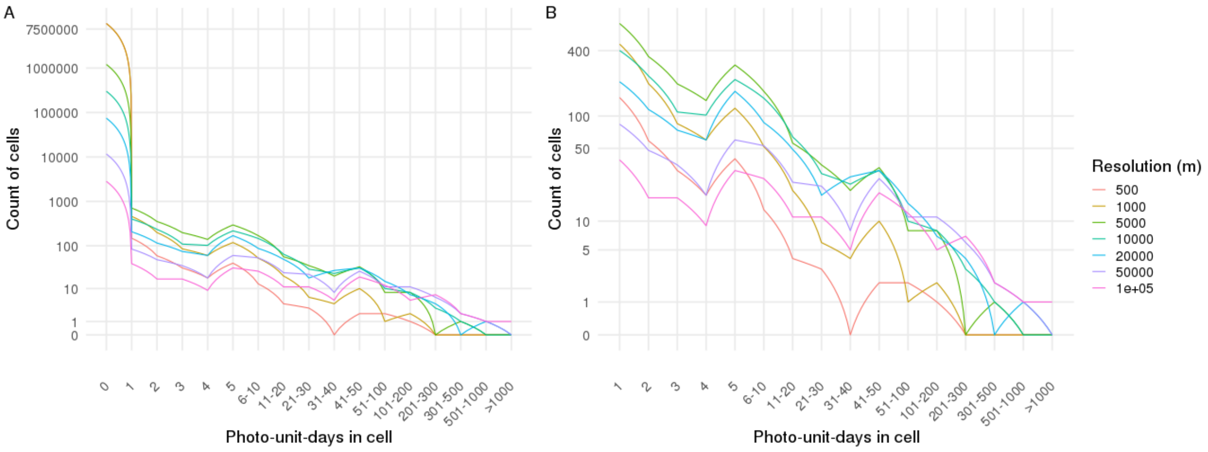

Figure S2.2 A. Histogram of Flickr photo density in the Arctic measured in photo-unit-days, relative to the resolution (cell diameter, m) of the raster at which point data was aggregated B. is a zoomed in view of A.
