# Supplementary material for: Quantifying tourism booms and the increasing footprint in the Arctic with social media data
Source: PLoS One. 2020 Jan 16;15(1):e0227189. doi: 10.1371/journal.pone.0227189 (PMC6964912; doi:10.1371/journal.pone.0227189)
Supplement: S3 Appendix — (PDF) [file pone.0227189.s003.pdf]

S3 Appendix: Global and Arctic Flickr trends

Table S3. Global and Arctic annual trends in Flickr use

Year	Number of photos uploaded to Flickr globally (billion photos; 10^8)		Proportional increase in global Flickr use from 2004		Number of photos uploaded to Flickr in the Arctic (absolute)		Percent traffic (n Arctic / n global, %)		Sample size *Global-bias corrected footprint*	
	Summer	*Winter*	*Summer*	*Winter*	*Summer*	*Winter*	*Summer*	*Winter*	*Summer*	*Winter*
2004	0.120	0.078	1.00	1.00	1118	287	0.0093	0.0037	1118	287
2005	0.328	0.204	2.75	2.61	2725	601	0.0083	0.0029	992	330
2006	0.683	0.449	5.71	5.74	7020	1377	0.0103	0.0031	1229	241
2007	1.339	0.806	11.19	10.30	15041	3710	0.0112	0.0046	1255	413
2008	1.604	1.235	13.41	15.78	21809	6090	0.0136	0.0049	1626	407
2009	1.747	1.424	14.61	18.20	24280	9004	0.0139	0.0063	1662	551
2010	1.791	1.439	14.98	18.39	33410	8935	0.0187	0.0062	2230	493
2011	1.948	1.553	16.29	19.84	42912	14421	0.0220	0.0093	2634	770
2012	1.891	1.533	15.81	19.59	64798	18201	0.0343	0.0119	4098	1013
2013	2.313	1.539	19.35	19.67	76900	37272	0.0332	0.0242	3975	2063
2014	2.228	1.789	18.64	22.85	68227	27500	0.0306	0.0154	3661	1223
2015	2.173	1.807	18.17	23.09	74354	37200	0.0342	0.0206	4091	1677
2016	2.039	1.707	17.05	21.81	81980	33966	0.0402	0.0199	4807	1460
2017	1.949	1.687	16.30	21.56	51631	30103	0.0265	0.0178	3168	1334

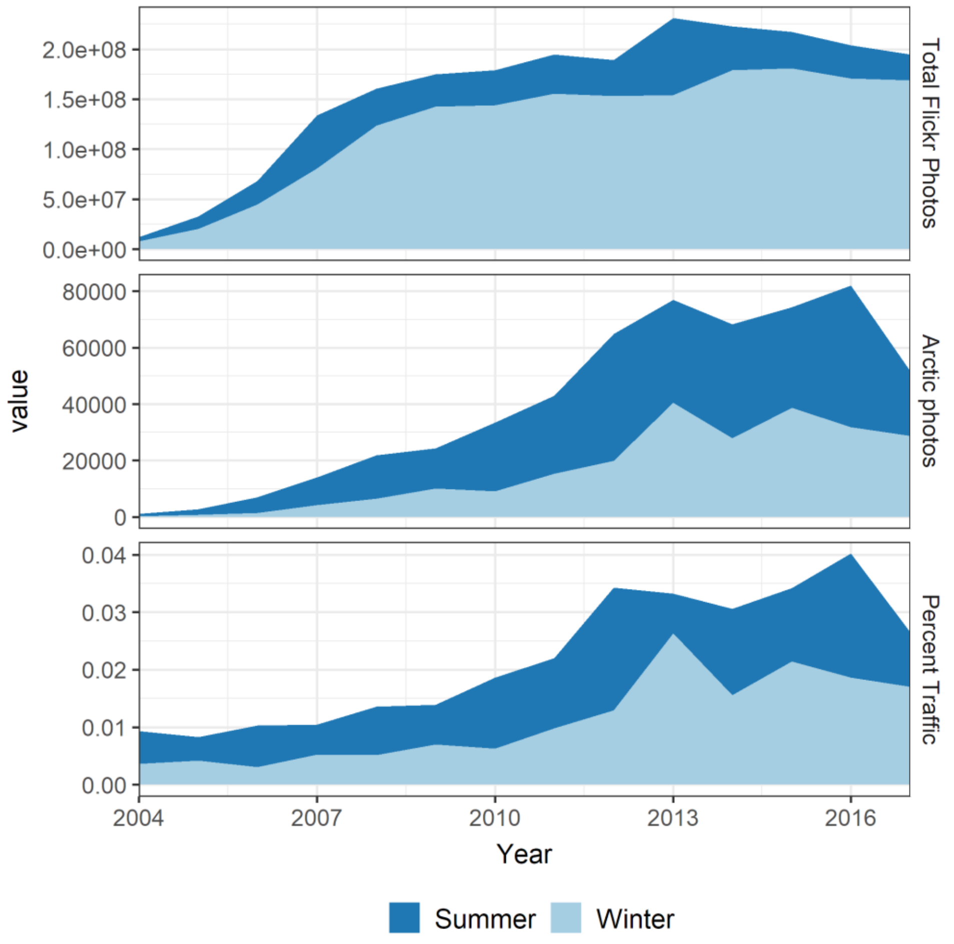

Figure S3 A. Annual number of photos uploaded to Flickr globally and B. in the Arctic C. Arctic photos make up an increasing percentage of global Flickr traffic.

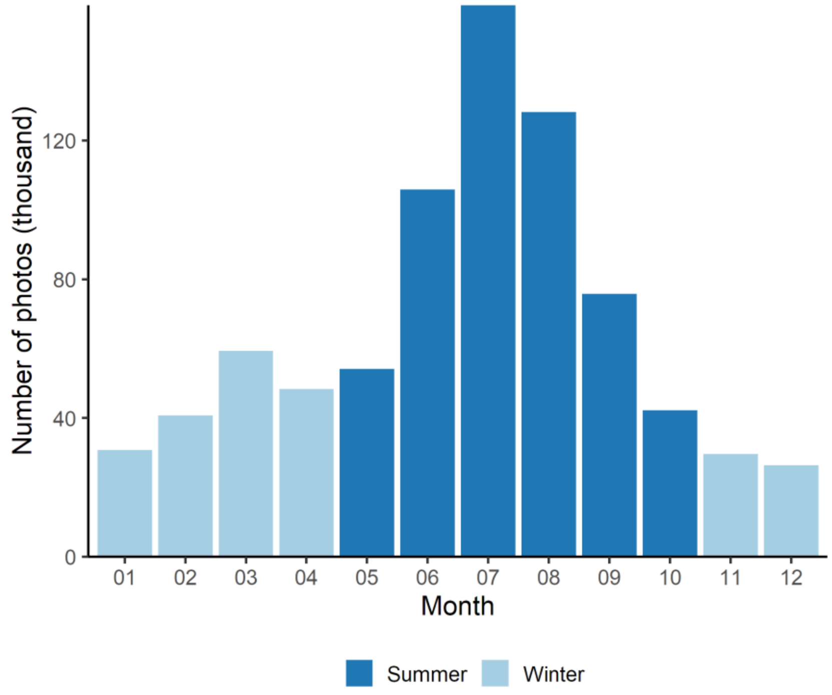

Figure S3 D: Number of photos uploaded to Flickr in the Arctic between 2004 and 2017, by month.
