# Supplementary material for: Quantifying tourism booms and the increasing footprint in the Arctic with social media data
Source: PLoS One. 2020 Jan 16;15(1):e0227189. doi: 10.1371/journal.pone.0227189 (PMC6964912; doi:10.1371/journal.pone.0227189)
Supplement: S4 Appendix — (PDF) [file pone.0227189.s004.pdf]

Appendix S4: Uncertainty around footprint estimates

The footprint under the methods *Equal sample size* and *Global bias-corrected* was estimated from subsamples of the Arctic Flickr dataset. The sample sizes are high, being in the thousands (*Equal sample size* winter) or tens of thousands (all other footprints) and thus we would expect the uncertainties around these estimates to be negligible. We performed 10 random draws and recalculated the summer and winter footprint using the *Equal sample size* methodology. The results, shown below, indicate that our findings are robust to the uncertainty introduced by sampling.

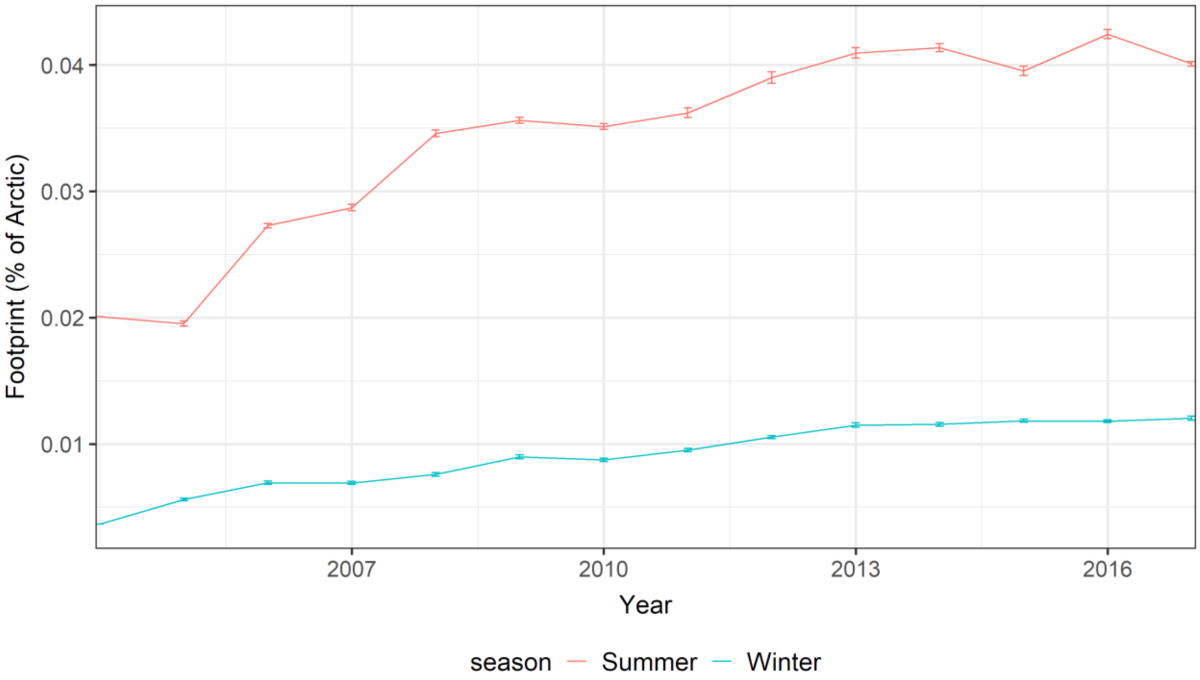

Figure S4 shows the mean of the footprint in summer (red line) and winter (blue line) across time, estimated the *Equal sample size* method. Error bars represent standard error around that mean estimated from 10 random draws.
