# Supplementary material for: Quantifying tourism booms and the increasing footprint in the Arctic with social media data
Source: PLoS One. 2020 Jan 16;15(1):e0227189. doi: 10.1371/journal.pone.0227189 (PMC6964912; doi:10.1371/journal.pone.0227189)
Supplement: S5 Appendix — (PDF) [file pone.0227189.s005.pdf]

S5 Appendix: GAM model diagnostics

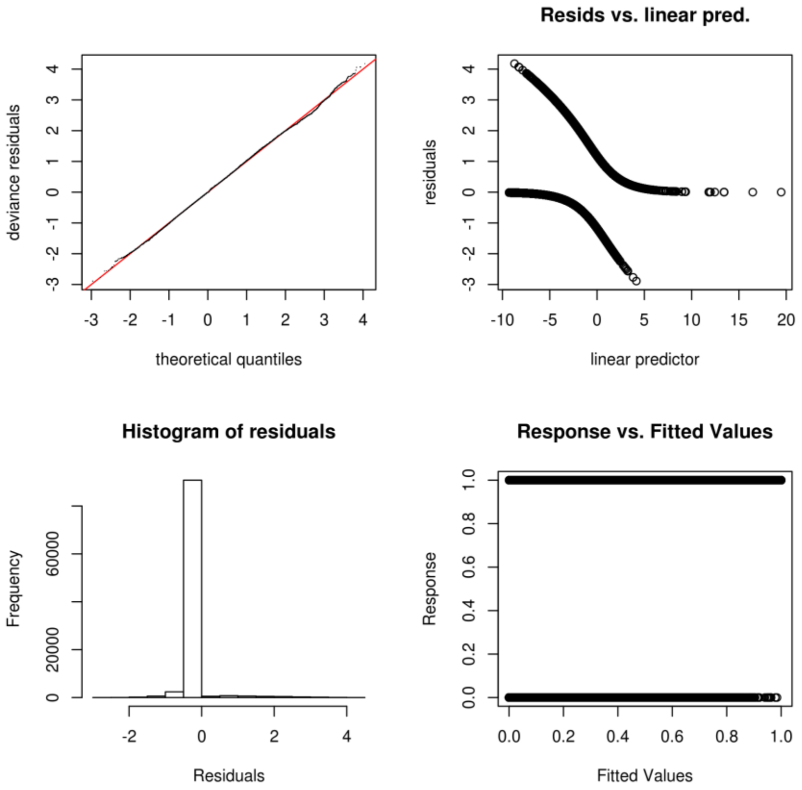

Figure S7A Model fit (full summer model)

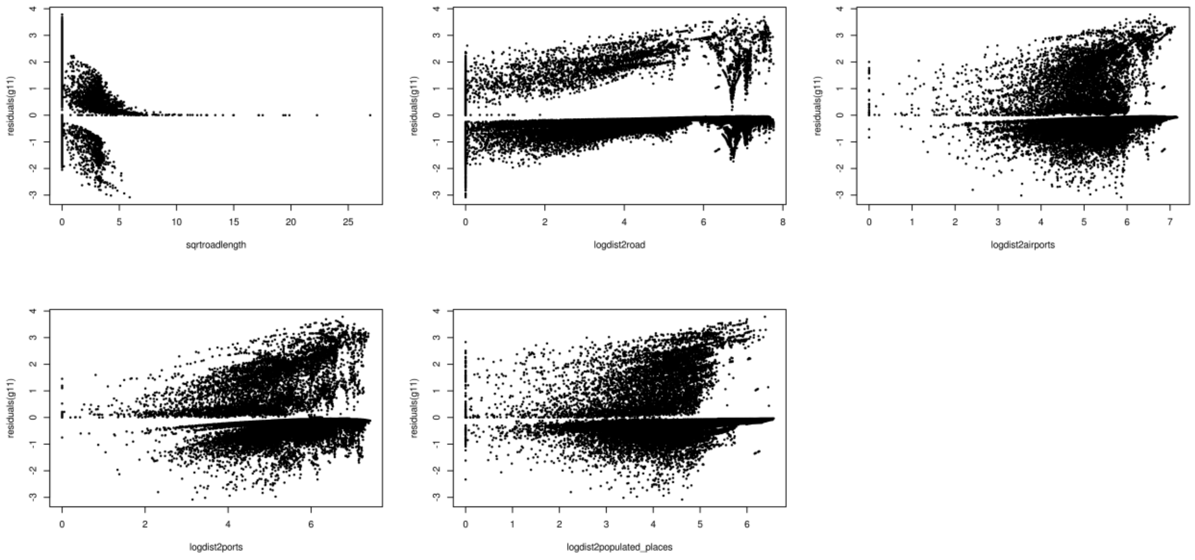

Figure S7B Model residuals of full summer model, plotted against accessibility variables (from left to right: square root of road length in cell, log distance to road, log distance to airport, log distance to ports, log distance to populated places).

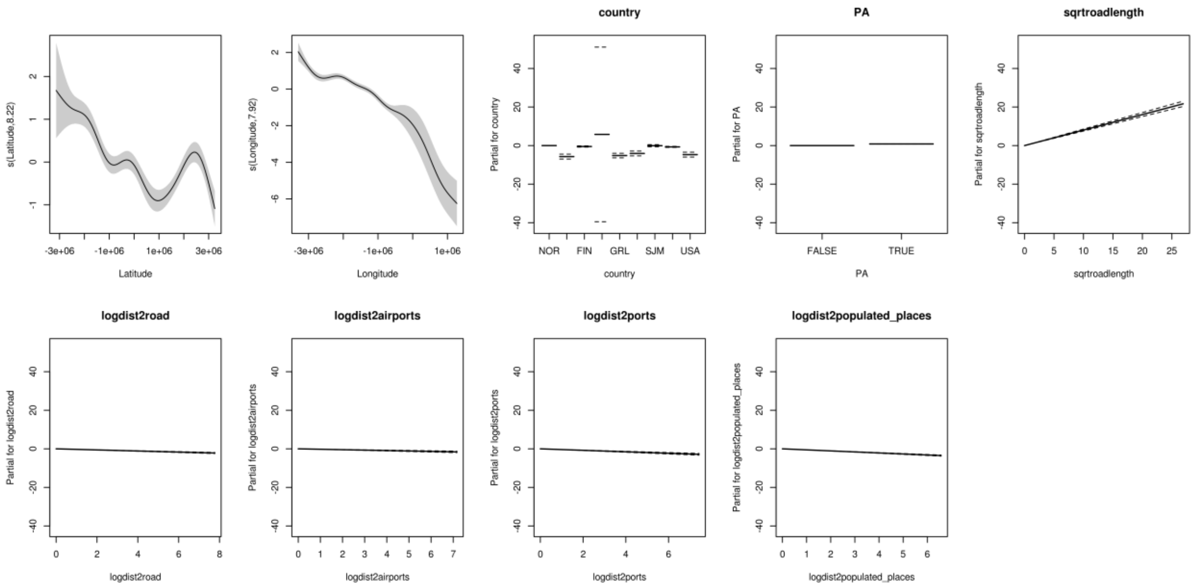

Figure S7C Partial plots of full summer model showing standard errors with mean (left to right: Latitude, longitude, country, protected areas, square root of road length in cell, log distance to road, log distance to airport, log distance to ports, log distance to populated places).

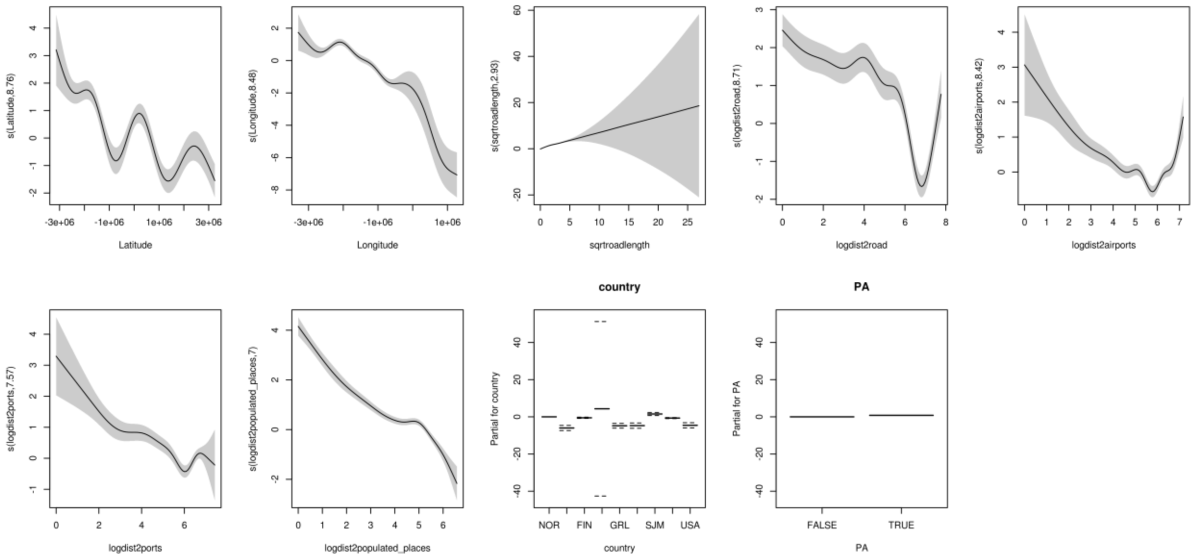

Figure S7D Partial plots of full summer model, with accessibility variables modelled with thin-plate splines, standard errors shown with mean (left to right: Latitude, longitude, square root of road length in cell, log distance to road, log distance to airport, log distance to ports, log distance to populated places, country, protected areas). Plot confirms that linear responses (shown above and reported in manuscript) are good approximations of the relationship between tourist footprint and accessibility.

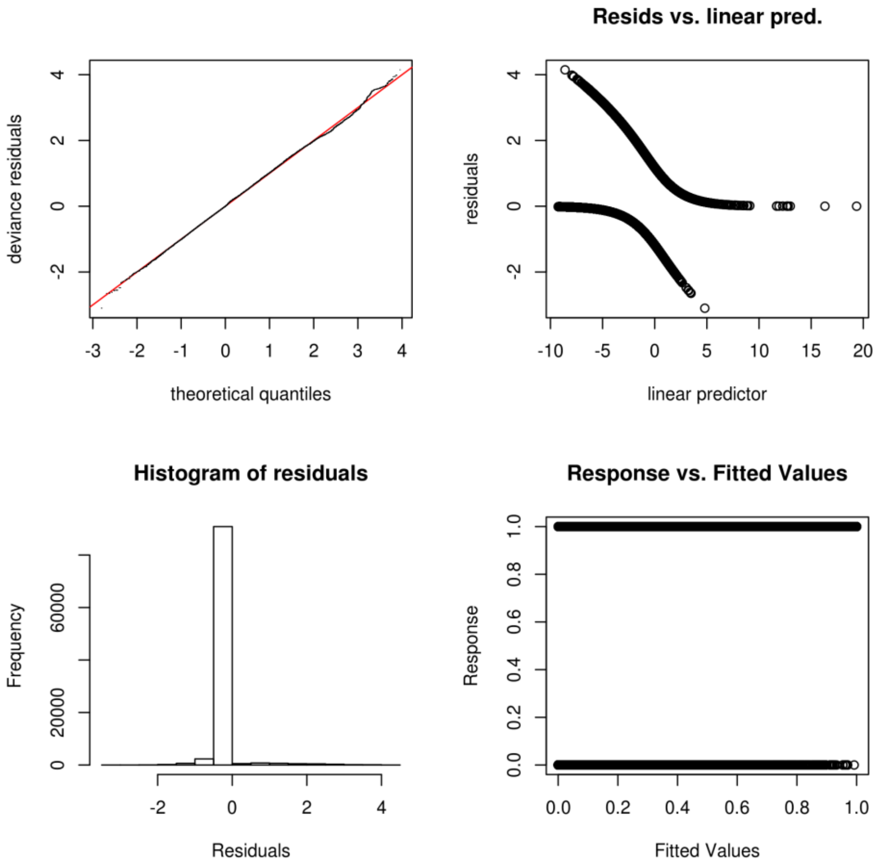

Figure S7E Model fit of winter model (without protected areas).

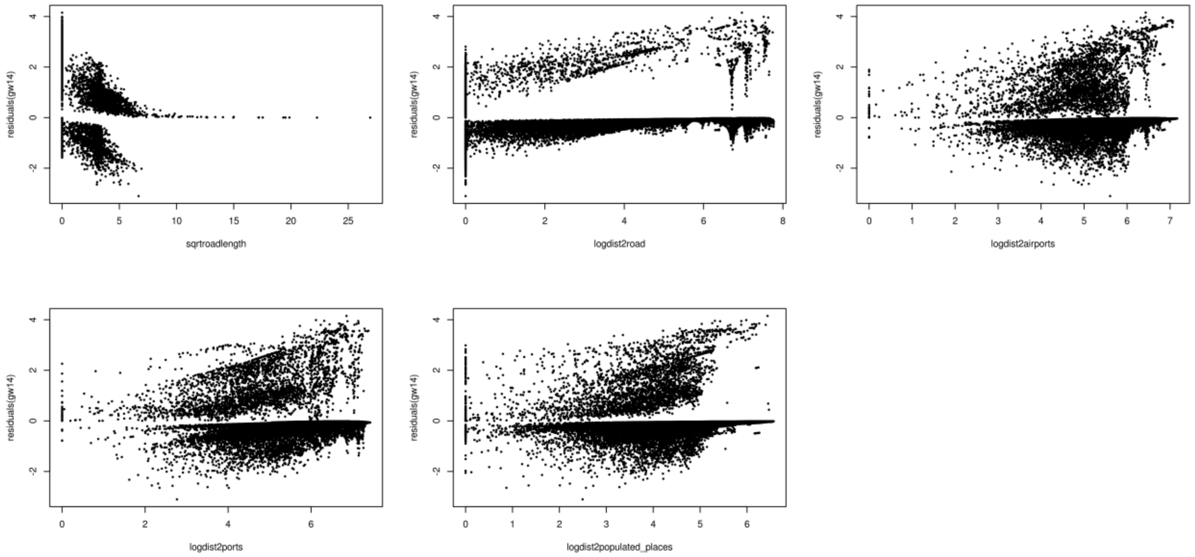

Figure S7F Model residuals of winter model (without protected areas), plotted against accessibility variables (from left to right: square root of road length in cell, log distance to road, log distance to airport, log distance to ports, log distance to populated places).

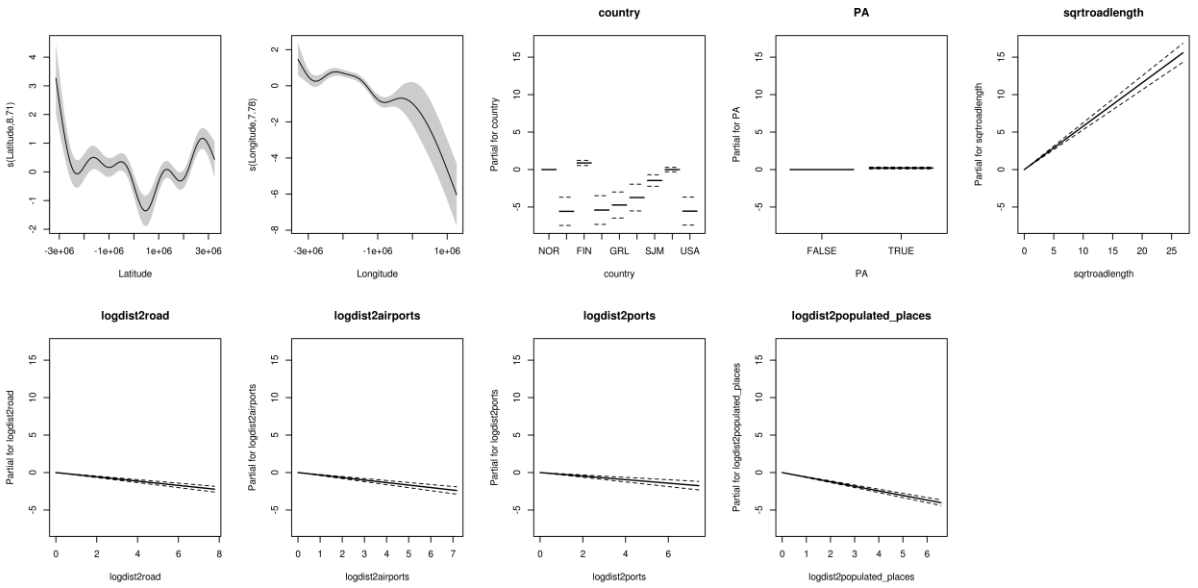

Figure S7G Partial plots of full winter model showing standard errors with mean (left to right: Latitude, longitude, country, protected areas, square root of road length in cell, log distance to road, log distance to airport, log distance to ports, log distance to populated places).

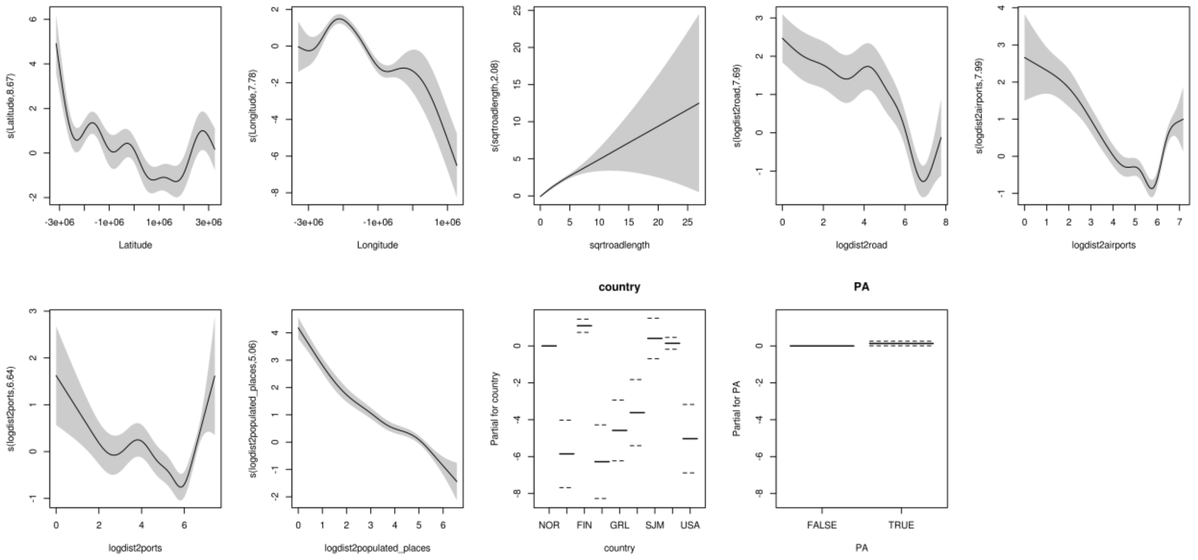

Figure S7H Partial plots of full winter model, with accessibility variables modelled with thin-plate splines, standard errors shown with mean (left to right: Latitude, longitude, square root of road length in cell, log distance to road, log distance to airport, log distance to ports, log distance to populated places, country, protected areas). Plot confirms that linear responses (shown above and reported in manuscript) are good approximations of the relationship between tourist footprint and accessibility.
