# Supplementary material for: Quantifying tourism booms and the increasing footprint in the Arctic with social media data
Source: PLoS One. 2020 Jan 16;15(1):e0227189. doi: 10.1371/journal.pone.0227189 (PMC6964912; doi:10.1371/journal.pone.0227189)
Supplement: S1 Fig — (PDF) [file pone.0227189.s006.pdf]

S1 Figure: Annual maps of tourism growth

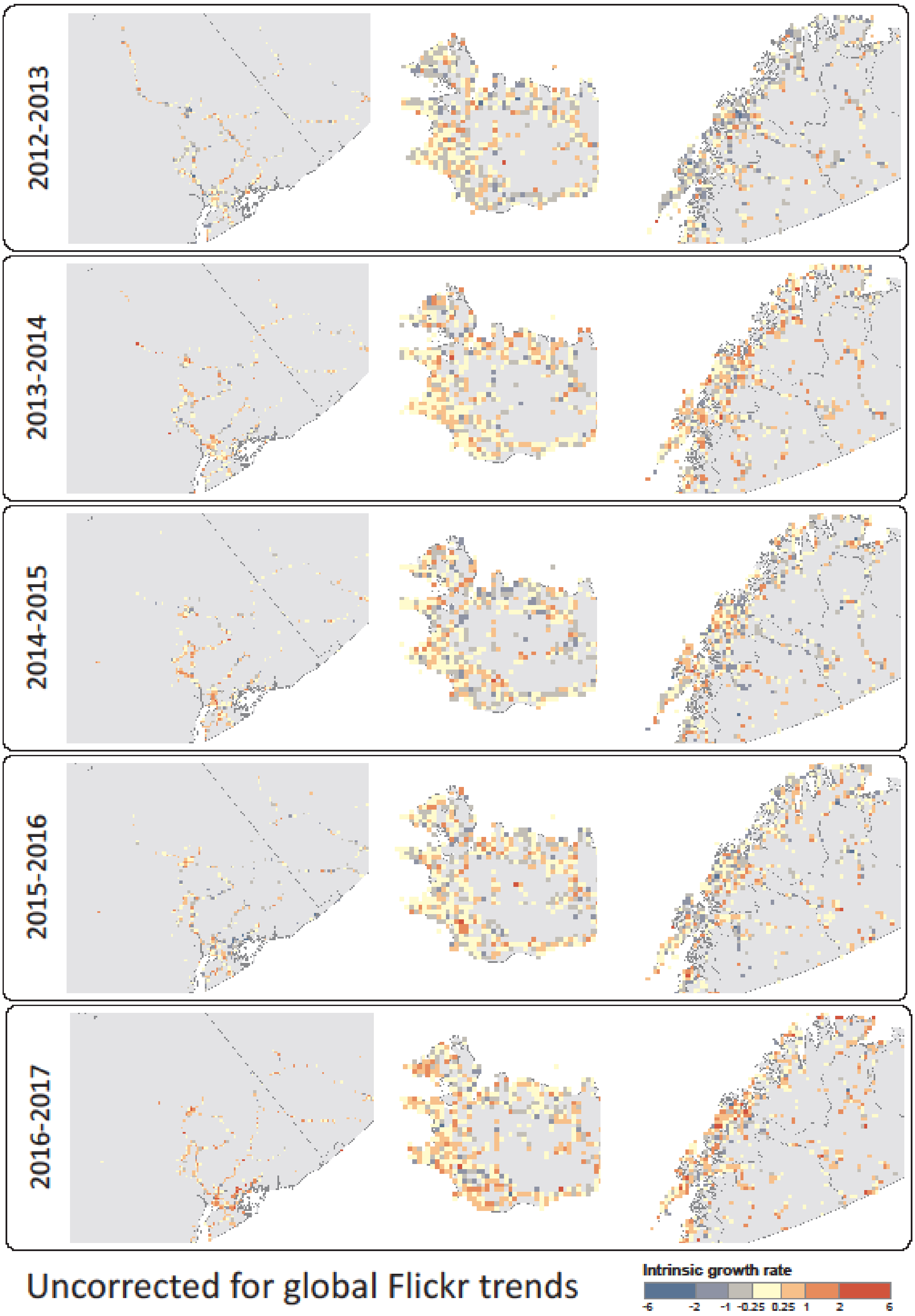

Figure S6A Annual growth in Artic tourism in North America, Iceland and northern Scandinavia between 2012 and 2017, estimated from the number of Flickr photo-unit-days in each 10 km cell. Intrinsic growth rate is the log of the photo-unit-days in year t2 minus the log of the photo-unit-days in year t1. Country borders are modified from Natural Earth CC PD.

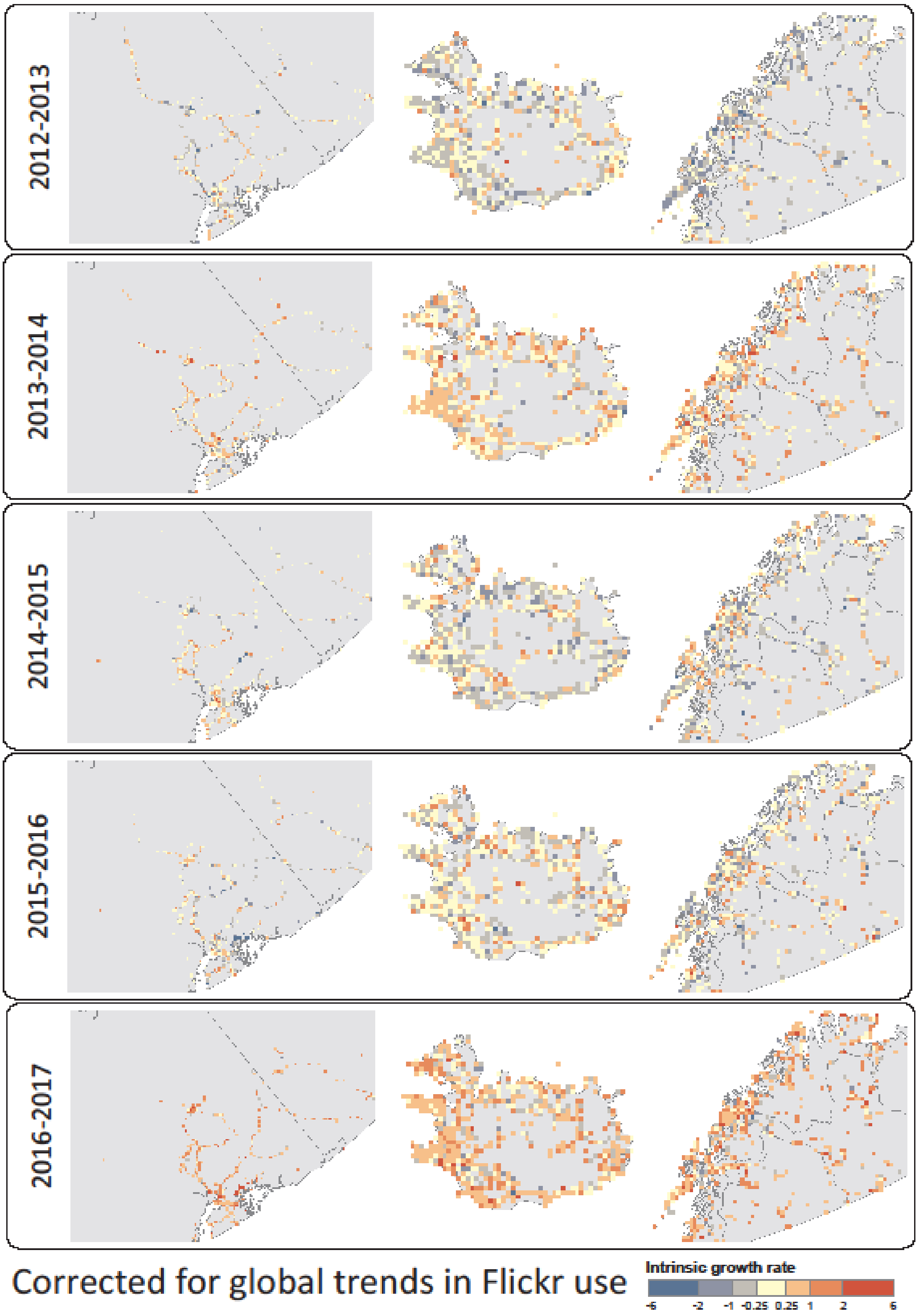

Figure S6B Annual growth in Artic tourism in North America, Iceland and northern Scandinavia between 2012 and 2017, estimated from the number of Flickr photo-unit-days in each 10 km cell, adjusted for global trends in Flickr use. The correction factor (Table S1.1) was estimated from the number of photos submitted to Flickr in the Arctic in a given year, divided by the number of photos submitted globally in that same year. The number of global Flickr users in any given year is unknown. Intrinsic growth rate was estimated as the log of (correction factor x photo-unit-days) in year t2 minus the log of (correction factor x photo-unit-days) in year t1. Country borders are modified from Natural Earth CC PD.

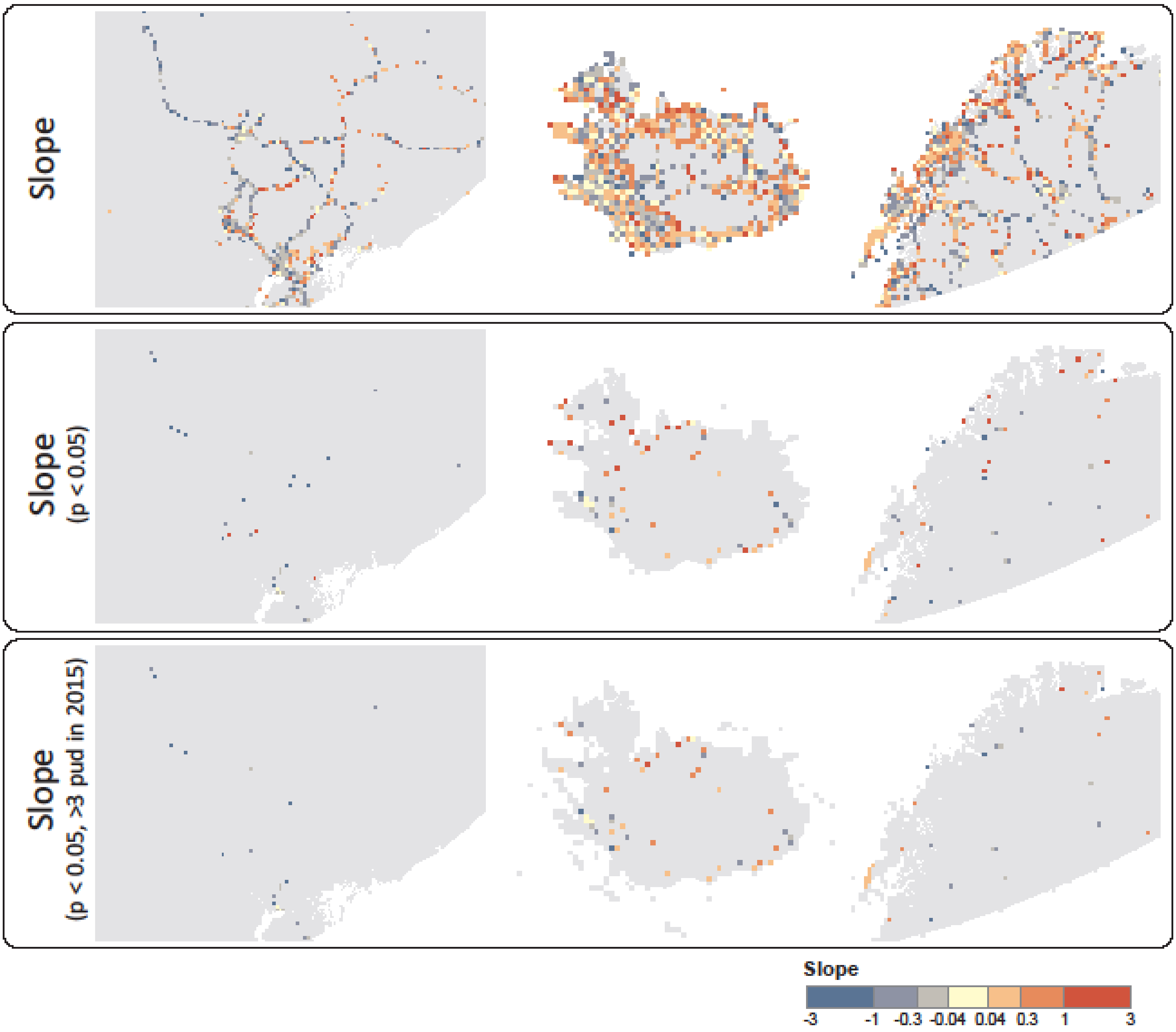

Figure S6C Booms and busts in Arctic tourism in North America, Iceland and northern Scandinavia between 2012 and 2017, estimated from uncorrected Flickr data. The top figure shows the slope of the temporal trend estimated from a linear model regressing photo-unit-days in each cell against time, in each cell that has been visited in at least two of the six years. The middle figure presents only the cells where the slope was significant at $p < 0.05$, and the bottom figure shows only cells where the slope was significant and the 10km cell was visited at least 3 photo-unit-days in 2015. Country borders are modified from Natural Earth CC PD.
