# Supplementary material for: Quantifying tourism booms and the increasing footprint in the Arctic with social media data
Source: PLoS One. 2020 Jan 16;15(1):e0227189. doi: 10.1371/journal.pone.0227189 (PMC6964912; doi:10.1371/journal.pone.0227189)
Supplement: S1 Table — (PDF) [file pone.0227189.s007.pdf]

S1 Table. Comparison with official visitor statistics

Table S5 Comparison between number of photos uploaded to Flickr and statistics on Arctic visitation

Metric	*Winter*	*Summer*	*Source*
Norway, seasonal visitation foreign and domestic visitors 2005 -2017, % (guest nights)	30.7 (12480417)	69.3 (28163172)	Statistics Norway (2018) Table 08401: Accommodation establishments total. Guest nights, by guests' country of residence (2005-2018). Available at https://www.ssb.no/en/statbank/list/overnatting/. Accessed 24/09/2018.
Norway 2004 -2017, photos	31.8	68.2	
Norway 2004 -2017, puds	36.3	63.7	
Finland, seasonal visitation foreign and domestic visitors 2004 -2017, % (guest nights)	58.1 (27604211)	41.8 (19843971)	Statistics Finland (2018) Table 002: Visitor arrivals and nights spend in hotels (2004-2017, North Ostrobothnia, Lapland, Kainuu). Available at https://pxnet2.stat.fi/PXWeb/pxweb/en/StatFin/ Accessed 25/09/2018.
Finland 2004 – 2017, photos	61.3	37.8	
Finland 2004 – 2017, puds	56.8	43.2	
Faroe Islands, 2016 seasonal visitation all accommodation types, % (bed nights)	26.1 (41724)	73.8 (11972)	Visit Faroe Islands (2016) Annual Report 2016. pp 6. Available at https://visitfaroeislands.com/content/uploads/2017/06/visitfaroeislandsannualreport2016en.pdf
Faroe Is. 2016, photos	28.3	71.7	
Faroe Is. 2016, puds	32.0	68.0	
Greenland, seasonal visitation foreign visitors 2004 -2017, %	27.6	72.4	Statistics Greenland (2018). Table TUXHOT: Overnight stays by region, time, month, unit and nationality (2004-2017, all regions). Available at http://bank.stat.gl/pxweb/en/Greenland/?rxid=TUXKAP25-09-2018+07%3a05%3a01 Accessed 25/09/2018.
Greenland, 2004-2017, photos	9.0	91.0	
Greenland, 2004-2017, puds	21.2	78.8	
